# Assessment of Safety and Effectiveness of the Extracorporeal Continuous-Flow Ventricular Assist Device (BR16010) Use as a Bridge-to-Decision Therapy for Severe Heart Failure or Refractory Cardiogenic Shock: Study Protocol for Single-Arm Non-randomized, Uncontrolled, and Investigator-Initiated Clinical Trial

**DOI:** 10.1007/s10557-018-6796-8

**Published:** 2018-06-08

**Authors:** Norihide Fukushima, Eisuke Tatsumi, Osamu Seguchi, Yoshiaki Takewa, Toshimitsu Hamasaki, Kaori Onda, Haruko Yamamoto, Teruyuki Hayashi, Tomoyuki Fujita, Junjiro Kobayashi

**Affiliations:** 1 Department of Transplant Medicine, National Cerebral and Cardiovascular Center, Suita, Osaka Japan; 2 Department of Artificial Organs, National Cerebral and Cardiovascular Center, Suita, Osaka Japan; 3 Department of Data Science, National Cerebral and Cardiovascular Center, Suita, Osaka Japan; 4 Center for Advancing Clinical and Translational Sciences, National Cerebral and Cardiovascular Center, Suita, Osaka Japan; 5 Deaprtment of Clinical Engineering, National Cerebral and Cardiovascular Center, Suita, Osaka Japan; 6 Department of Cardiac Surgery, National Cerebral and Cardiovascular Center, Suita, Osaka Japan

**Keywords:** Continous-flow ventricular assist device, Bridge to decision, Advanced heart failure, Investigator-initiated clinical trial

## Abstract

**Background:**

The management of heart failure patients presenting in a moribund state remains challenging, despite significant advances in the field of ventricular assist systems. Bridge to decision involves using temporary devices to stabilize the hemodynamic state of such patients while further assessment is performed and a decision can be made regarding patient management. The purpose of this study (NCVC-BTD_01, National Cerebral and Cardiovascular Center-Bridge to Dicision_01) is to assess the safety and effectiveness of the newly developed extracorporeal continuous-flow ventricular assist system employing a disposable centrifugal pump with a hydrodynamically levitated bearing (BR16010) use as a bridge-to-decision therapy for patients with severe heart failure or refractory cardiogenic shock.

**Method/Design:**

NCVC-BTD_01 is a single-center, single-arm, open-label, exploratory, medical device, investigator-initiated clinical study. It is conducted at the National Cerebral and Cardiovascular Center in Japan. A total of nine patients will be enrolled in the study. The study was planned using Simon’s minimax two-stage phase design. The primary endpoint is a composite of survival free of device-related serious adverse events and complications during device support. For left ventricular assistance, withdrawal of a trial device due to cardiac function recovery or exchange to other ventricular assist devices (VADs) for the purpose of bridge to transplantation (BTT) during 30 days after implantation will be considered study successes. For right ventricular assistance, withdrawal of tal device due to right ventricular function recovery within 30 days after implantation will be considered a study success. Secondary objectives include changes in brain natriuretic peptide levels (7 days after implantation of a trial device and the day of withdrawal of a trial device), period of mechanical ventricular support, changes in left ventricular ejection fraction (7 days after implantation of a trial device and the day of withdrawal of a trial device), and changes in left ventricular diastolic dimension (7 days after implantation of a trial device and the day of withdrawal of a trial device).

**Ethics and Dissemination:**

We will disseminate the findings through regional, national, and international conferences and through peer-reviewed journals.

**Trial Registration:**

UMIN Clinical Trials Registry (UMIN-CTR; R000033243) registered on 8 September 2017.

## Background

Implantation of ventricular assist devices (VADs) has become an indispensable alternative for the treatment of patients with severe end-stage heart failure [1–3]. However, despite recent advancements in the field of VADs, the outcome remains poor for patients classified as interagency registry for mechanically assisted circulatory support (INTERMACS) profile 1 especially with severe respiratory, renal, and hepatic failure [4]. Patients with serious low cardiac output syndrome require an adequate amount of blood flow to recover from severe multi-organ failure. The use of temporary devices such as extracorporeal VAD provides a solution to establish adequate blood flow and stabilize the hemodynamic state of the patients while further assessment of the clinical condition is being performed and a process referred to as bridge to decision (BTD), when it is unclear whether the patient’s heart will recover or whether the patient will need alternative, longer-term therapy or transplantation [5–8]. In terms of cost-effectiveness, an extracorporeal continuous-flow VAD that uses a disposable pump is desirable for BTD [9]. Recently, some clinicians have reported using a conventional disposable centrifugal pump and a cannula for cardiopulmonary bypass (CPB) for BTD [10, 11]. However, in conventional pumps with contact bearings, thrombus formation occurs within just a few days because of blood cell destruction and blood coagulation caused by mechanical wear and heat generation. A blood pump system with longer durability and superior hemocompatibility than conventional pump for cardiopulmonary bypass, and lower cost than implantable VADs is required that is able to provide enough time for decision-making without the risk of system replacement or system-related complications. Recent developments in the field of blood pumps have focused on noncontact bearing systems with hydrodynamically or magnetically levitated bearings, which have demonstrated excellent blood compatibility and low hemolytic effect, ensuring long-term durability of the VADs [12–15]. As regards an implantable left VAD (LVAD), HeartMate 3 LVAD was invented as a fully magnetically levitated centrifugal-flow chronic LVAD and Netuka et al. [15] showed that HeartMate 3 was safe, with high 30-day and 6-month survival rates, a favorable adverse event profile, and improved quality of life and functional status. The prospective, randomized Multicenter Study of MagLev Technology in Patients Undergoing Mechanical Circulatory Support Therapy with HeartMate 3 (MOMENTUM 3) clinical trial has been carried out to evaluate the safety and effectiveness of the HeartMate 3 LVAD by demonstrating non-inferiority to the HeartMate II LVAD (also St. Jude Medical, Inc.) [16].

As regards extracorporeal VAD, there is no approved centrifugal-flow pump for LVAD use in the world. The Levitronix® CentriMag® ventricular assist system (VAS) (Levitronix LLC; Waltham, MA) is a magnetically levitated centrifugal-flow pump that can be clinically applied as LVAD and right VAD (RVAD) in USA [6, 8]. Although the Levitronix CentriMag is authorized by federal law to provide temporary circulatory support for up to 30 days for patients in cardiogenic shock due to acute right ventricular failure, the effectiveness of this device for this use has not been demonstrated and distribution of this device is restricted to use by or on the order of a physician. When used for left or biventricular support for periods over 6 h, the CentriMag VAS is an investigational device limited by federal (USA) law to investigational use. This device will be used for up to 30 days to support one or both sides of the heart as a bridge to decision, when it is unclear whether the patient’s heart will recover or whether the patient will need alternative, longer-term therapy or transplantation. This prospective, non-randomized trial will enroll a total of 30 patients at up to 25 clinical centers. The CentriMag Pivotal Trial will be conducted jointly by Thoratec and Levitronix.

The core components of the CentriMag VAS consist of a continuous-flow centrifugal blood pump, a primary console and motor, a flow probe, a backup console and motor, a tubing, and cannulas. The pump, which has a priming volume of 31 mL, can be operated up to a maximum speed of 5500 rpm while generating up to 9.9 L/min flow [17]. When the pump is inserted into the motor and activated, the internal impeller is electromagnetically levitated and centered, eliminating the need for shafts, seals, and bearings in the pump. Shafts, seals, and bearings are the sites typically responsible for hemolysis, thrombus, and particle formation. Large gaps between the impeller and the pump housing are designed to minimize potential shear forces on blood cells, thereby allowing high blood flow rate with minimal hemolysis. Utilizing magnetic levitation to suspend and spin the impeller reduces friction in the pump and eliminates the point source of friction (bearing) resulting in minimal heat and wear of the pump components.

Our center developed a new extracorporeal continuous-flow temporary LVAD that uses a novel disposable centrifugal blood pump with a hydrodynamically levitated bearing (HLB). As ensuring the performance and safety of the VAD as a whole is important, the combination of the pump, cannulas, connectors, and circuits was well designed. An HLB utilizes hydrodynamic pressure at the small gaps between the rotating part (the impeller) and the stationary part (the casing wall) to counter mechanical loads, and this pressure, in a general sense, is dependent on the rotational speed (RS). There remains a possibility of mechanical contact between the rotating part and the stationary part when the blood pump is operated at a low speed. The effectiveness and safety of the pump in the setting of extracorporeal membrane oxygenation have been reported [18], in which the afterload of the blood pump is assumed to be higher than that in LVAD cases because of small cannula diameters and the hydraulic resistance of oxygenators. In the goat model, this newly developed temporary LVAD demonstrated consistent and satisfactory hemodynamic performance and hemocompatibility over the course of a 30-day experiment [19]. The outcomes of this previous study have served to establish the optimal composition and design of our new LVAD for BTD and this newly developed LVAD represents a promising tool for BTD in critically ill patients.

The purpose of this study (NCVC-BTD_01, National Cerebral and Cardiovascular Center-Bridge to Dicision_01) is to assess the safety and effectiveness of the extracorporeal continuous-flow VAD (BR16010) use as a bridge-to-decision therapy for patients with severe heart failure or refractory cardiogenic shock.

## Methods and Analysis

### Study Design

NCVC-BTD_01 is a single-center, single-arm, open-label, exploratory, medical device, investigator-initiated clinical study. It is conducted at the National Cerebral and Cardiovascular Center in Japan.

### Study Period

Enrollment of study participants started in October 2017 and will end in July 2018; then, results of this study will be published.

### Sample Size

A total of nine patients will be enrolled in the study. The study was planned using Simon’s minimax two-stage phase design [20]. The hypothesis to be tested was H0: P < P0 vs H1: P > P1, where P is the probability of mortality at 30 days, accepting a false-positive rate (*α*) ≤ 10% and a false-negative rate (*β*) ≤ 10%, and P0 was set to 50% and P1 was set to 90%. These parameters lead to a two-stage design with the first stage of four patients stopping if two or fewer survive, or continuing accrual to a total of nine patients, declaring success if more than seven patients survive.

### Study Objectives and Endpoints

The primary objective of this first-in-human trial (NCVC-BTD_01) is to assess the safety and effectiveness of the BR16010 to support one or both sides of the heart as a BTD VAD for patients with severe heart failure or refractory cardiogenic shock refractory to optimal medical management, standard surgical procedures, or mechanical circulatory supports [e.g., intra-aortic balloon pumping (IABP), ventriculo-arterial bypass (VA bypass), and percutaneous cardiopulmonary support (PCPS)]. The study had a single set of entry criteria for patients designated for bridge to recovery (BTR) or bridge to transplantation (BTT).

The primary endpoint is a composite of survival free of device-related serious adverse events and complications during device support. For left ventricular assistance, withdrawal of a trial device due to cardiac function recovery or exchange to other VADs for the purpose of BTT during 30 days after implantation will be considered study successes. For right ventricular assistance, withdrawal of trial device due to right ventricular function recovery within 30 days after implantation will be considered a study success.

Secondary endpoints include changes in brain natriuretic peptide (BNP) levels (7 days after implantation of a trial device and the day of withdrawal of a trial device), period of mechanical ventricular support, changes in left ventricular ejection fraction (LVEF) (7 days after implantation of a trial device and the day of withdrawal of a trial device), and changes in left ventricular diastolic dimension (LVDd) (7 days after implantation of a trial device and the day of withdrawal of a trial device).

## Device Description

### Novel Centrifugal Blood Pump with HLB

**Fig. 1:**
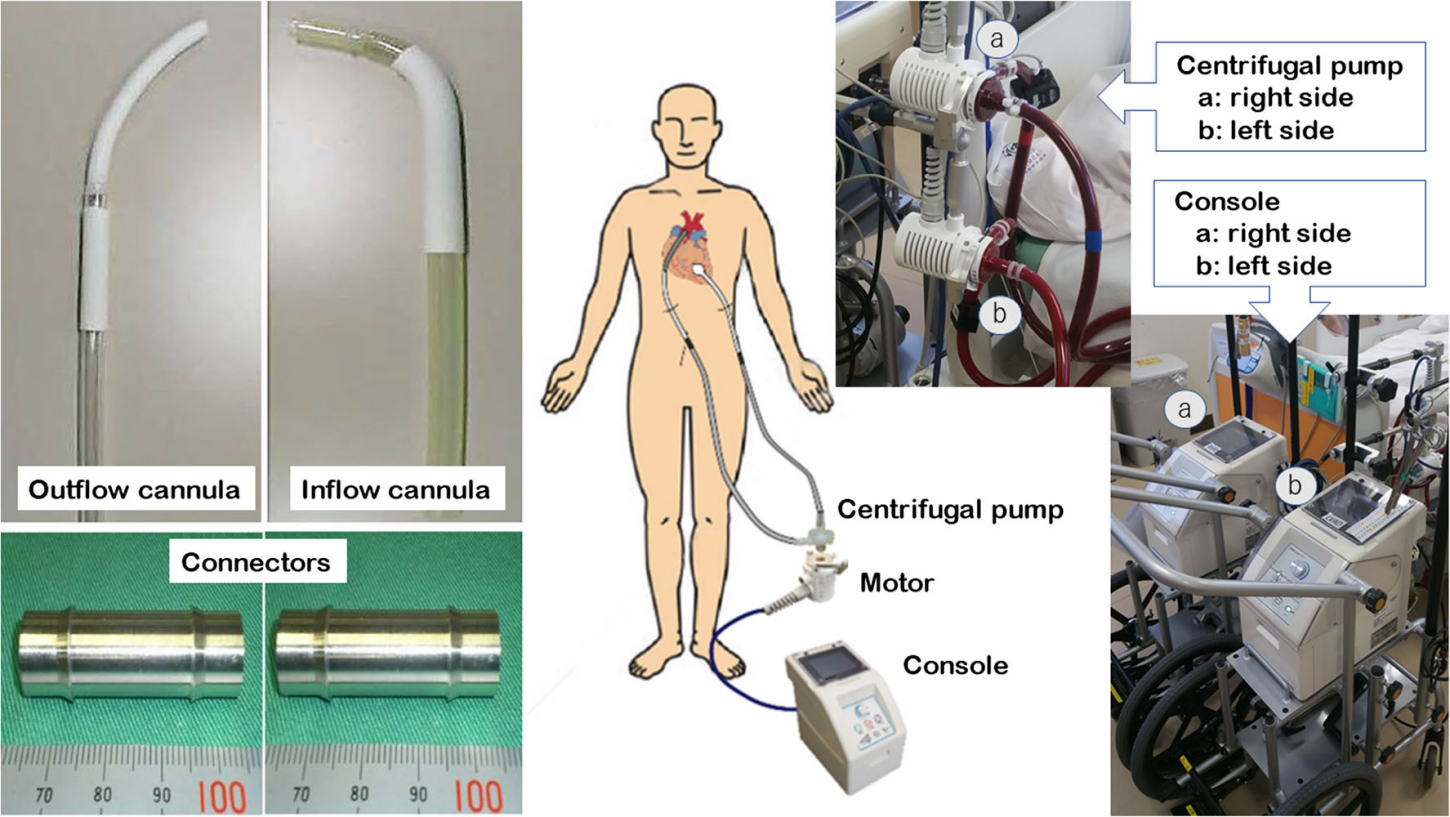
Components of the new temporary left ventricular assist system developed in this study and overview of the clinical setup

**Fig. 2:**
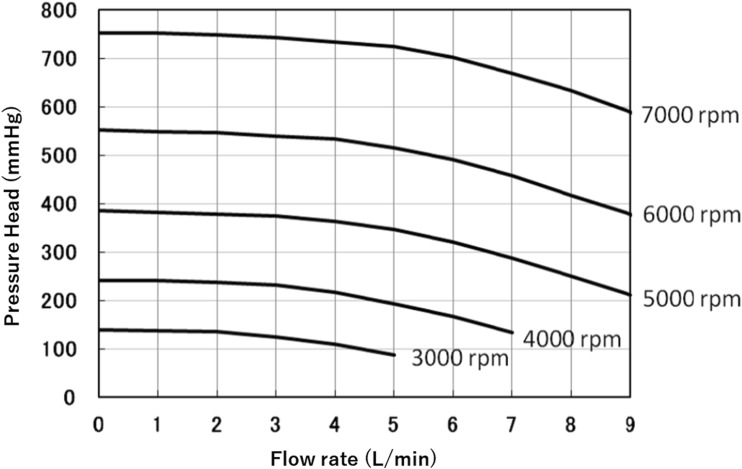
The H-Q curve of the hydrodynamically levitated bearing pump of the new temporary left ventricular assist system developed in this study

The newly developed temporary VAD consists of a disposable centrifugal pump with an HLB. The priming volume of the pump is 18 mL. The pump-and-motor unit is 64 mm in diameter, 131 mm in height, and weighs 635 g (Fig. 1). The impeller is embedded in the stationary casing and rotates with a narrow clearance at both ends. The blood film in this clearance serves as the journal bearing, which generates sufficient pressure to sustain the impeller and avoid mechanical contact with the stationary part. The pump, which was previously described elsewhere [18], achieved a flow rate of 5.0 L min against a pressure head of 400 mmHg at 5150 rpm of RS (Fig. 2). The performance of the pump can ensure a sufficiently high flow rate even in patients with severe heart failure. However, for left ventricular assist, the operation conditions require a flow rate of 5.0 L min against a pressure head of approximately 100 mmHg (without circuit resistance), which implies decreasing the RS of the pump. On the other hand, maintaining a higher RS was recommended to achieve stable operation of the pump with the HLB. Therefore, it was necessary to ensure that the RS required for left ventricular assist can be maintained under stable pump operation. We built several mock circuits to investigate the contribution of the resistance of the cannulas and extracorporeal circuit, and identified candidates expected to provide adequate resistance for maintaining the RS necessary for stable pump operation. In addition, we performed in vitro hemolysis testing of the pump under the condition assumed for use in left ventricular assist. The pumps were operated at several rotational speeds, maintaining a mean flow rate of 5.0 L min for 4 h. The calculated normalized indexes of hemolysis (NIH) were 0.0003 at 3000 rpm (*n* = 1) and 0.0042 at 2500 rpm (*n* = 1). The NIH was elevated between 2500 and 3000 rpm. Based on these results, we determined that the lowest RS acceptable to avoid hemolysis was 3000 rpm in consideration of securing sufficient range of safe.

### Cannula

A 1/2-in. flexible polyvinyl chloride (PVC) tube with a hard tip (35 mm in length) and a 12-mm diameter vascular prosthesis attached to a flexible PVC tube were used as the inflow and outflow conduits, respectively (Fig. 1).

### Extracorporeal Circuit and Metallic Connector

The extracorporeal circuit, which ran between the pump head and the cannulas, consisted of 70-cm-long and 3/8-in.-wide PVC tubes. Custom-made metallic connectors were used for connecting the cannulas and extracorporeal circuit, to minimize thrombogenesis (Fig. 1). All the blood-contacting surfaces of the circuit were coated with T-NCVC heparin coating (TOYOBO, Osaka, Japan).

## Patient Enrollment and Study Schedule

Inclusion and exclusion criteria are shown in Table 1. Patients with severe heart failure or refractory cardiogenic shock refractory to optimal medical management, standard surgical procedures, or mechanical circulatory supports (e.g., IABP, VA bypass, and PCPS) are candidates for the study. All patients are required to provide written informed consent. Considering that BTD is a medical emergency, written informed consent of the patient’s next of kin is accepted in patients who are unconscious by therapeutic reason. Patients are notified at enrollment of their freedom to abandon the study at any time without consequences.

**Table 1:** Inclusion and exclusion criteria

Inclusion criteria	
(1) Serious heart failure or refractory cardiogenic shock due to the following diseases, which does not respond to optimal medication and mechanical support	
- Low cardiac output syndrome due to cardiac disorders (idiopathic, secondary or ischemic cardiomyopathy, or myocarditis)	
- Post-acute myocardial infarction circulatory failures (including mechanical complication or refractory arrhythmia)	
- Difficulty in withdrawal of mechanical circulatory support	
- Postoperative low cardiac output syndrome (including refractory arrhythmia)	
- Other cardiogenic circulatory failures (including refractory arrhythmia)	
(2) Fully recovery is not expected with optimal medication, standard surgical procedures, and mechanical circulatory support	
(3) Body weight not lower than 10 kg at the time of informed consent	
(4) NYHA IV (Ross IV for children less than 7 years old) and at least one of the following conditions	
- INTERMACS/JMACS profile status 1 or 1A	
- INTERMACS/JMACS profile status 2 or 2A	
- Currently supported with extracorporeal membrane oxygenation (ECMO) or percutaneous cardiopulmonary support device (PCPS)	
(5) Written informed consent of the patient or his/her relatives	
Exclusion Criteria	
(1) Unfavorable or technically challenging cardiac anatomy	
(2) Evidence of irreversible hepatic disease (except when the primary investigator deems it as a sign of acute heart failure)	
(3) Evidence of irreversible renal disease (except when the primary investigator deems it as a sign of acute heart failure)	
(4) Evidence of irreversible intrinsic respiratory disease (e.g., chronic pulmonary disease, acute respiratory distress syndrome) which needs mechanical respiratory support (except when the primary investigator deems it as a sign of acute heart failure)	
(5) Contraindicated for anticoagulation	
(6) Difficulty of 30-day observation is anticipated	
(7) Pregnant or suspected pregnant at time of study entry	
(8) Participating in another clinical trial at time of study entry	
(9) Deemed unsuitable by the primary investigator for other reasons	

**Fig. 3:**
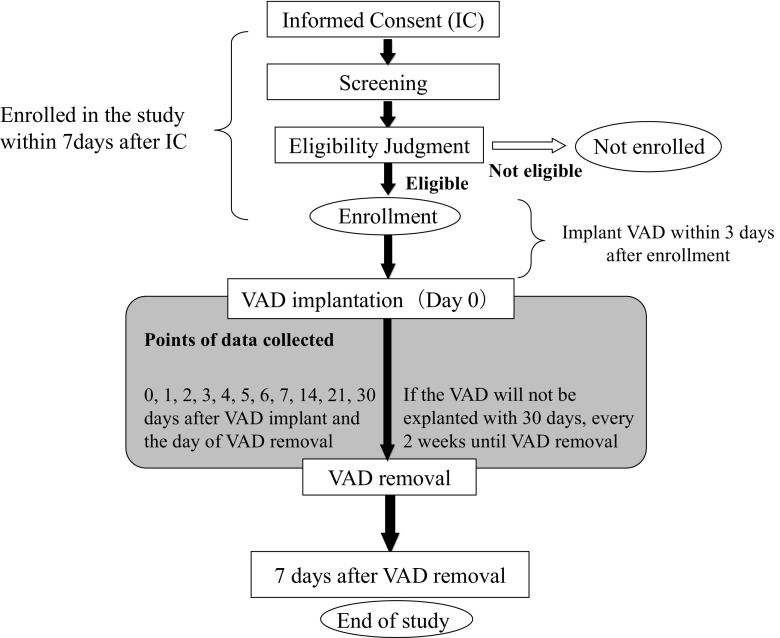
Summary of the study design

The trial VAD is implanted within 3 days after study entry. Patients were followed to the primary endpoint at 30 days after implantation of trial VAD or other outcomes (transplant, explant, or death), whichever occurred first. Patients will be followed for 7 days after removal or until other outcomes. For enhancing patient safety, the enrollment is held after the fourth patient is registered until the independent safety board confirms the 7-day safety for the patients. A summary of the study design is shown in Fig. 3. The schedule of data to be collected is presented in Table 2.

**Table 2:** Data to be collected as part to of this study

Variables	Screening (baseline)	1–7 days	14 days	21 days	30 days	Every 2 weeks after 30 days	At VAD removal	7 days after removal
Patient information								
Basic characteristics	○							
Vital signs	○	○	○	○	○	○	○	
Image examinations								
Electrocardiogram	○	○	○	○	○	○	○	
Ultra sound cardiography	○	○	○					
Chest X-ray	○							
Brain CT scan	○							
Blood examinations								
Blood cell counts, electrolytes, chemistry, coagulation, etc.	○	○	○	○	○	○	○	
Heart function								
NYHA classification (or Ross classification)	○	○	○					

*CT* computer tomography, *NYHA* New York Heart Association

### Independent Safety Evaluation Board

The independent safety evaluation board is comprised of three individuals not involved in the study conduct who have expertise in multiple disciplines, including a cardiologist, a nephrologist, and a neurologist. All serious adverse events are reported to and discussed by the independent safety board, and the 7-day safety of the first four patients is evaluated.

### Data Management, Monitoring, and Auditing

The system for electronic data capture and data management is validated to meet the regulatory requirements. On-site monitoring including source document verification and audit is planned.

### Statistical Analysis

Analyses will be done in all patients recruited into the study as a primary analysis population. Patient demographic data, safety, and efficacy endpoints will be analyzed descriptively: continuous data were expressed as mean with SD or median with range (minimum and maximum), whereas categorical data were expressed as numbers with percentages. All reported confidence intervals will be two-sided with 95% confidence level, and especially for binary endpoints, an exact CI for the proportion is calculated using the Clopper-Pearson method. All analyses are performed according to a prespecified statistical analysis plan using SAS V.9.3 or later (SAS Institute). The statistical analysis plan will be prepared separately and finalized before database-locking.

### Ethics and Dissemination of Results

The study is conducted in compliance with the Declaration of Helsinki, International Conference on Harmonization/Good Clinical Practices, and the International Organization for Standardization of medical devices for human subjects, known as 14155:2011, and in accordance with country-specific requirements.

The study protocol (NCVC-BTD_01) has been reviewed and approved by National Cerebral and Cardiovascular Center Institutional Review Board (approved on 7/30/2017). A clinical trial notification as a new medical device, much like a US investigational device exemption application, was accepted by the Japanese regulatory agency before it started. The study was registered in UMIN Clinical Trials Registry (UMIN-CTR; R000033243). After the first patient was enrolled on October 6, 2017, four patients have been enrolled in this study at the end of January 2018.

The results of this study will be submitted for publication in peer-reviewed journals.
